# Hydroalcoholic Extract of *Ferula aucheri* Shows Anti-depressant Effect Against Lipopolysaccharide-Induced Depression in Mice: Involvement of NF-κB and TLR4 Signaling Pathway

**DOI:** 10.5812/ijpr-166242

**Published:** 2026-03-02

**Authors:** Mohammad Mehdi Gravandi, Maryam Naseri, Nasim Jamshidi, Samira Shirooie

**Affiliations:** 1 Student Research Committee, Kermanshah University of Medical Sciences, Kermanshah, Iran; 2 Pharmaceutical Sciences Research Center, Health Institute, Kermanshah University of Medical Sciences, Kermanshah, Iran

**Keywords:** *Ferula aucheri*, Neuroinflammation, Cytokines, Depression, Mice

## Abstract

**Background:**

Depression is one of the most common neuropsychiatric disorders worldwide. Lipopolysaccharide (LPS), a bacterial endotoxin, induces depressive-like behaviors by activating microglia and promoting the release of pro-inflammatory mediators, including nitric oxide, eicosanoids, and various cytokines. Owing to its rich content of flavonoids, phenolic compounds, and terpenoids, *Ferula aucheri* possesses potent antioxidant and anti-inflammatory properties, which may contribute to its potential as an alternative treatment for depression.

**Objectives:**

This study aimed to evaluate the antidepressant-like effects of the hydroalcoholic extract of *F. aucheri* on LPS-induced depression in mice.

**Methods:**

After preparation of the hydroalcoholic extract of *F. aucheri*, 30 mice were randomly divided into five groups: (1) Control; (2) LPS (1 mg/kg, i.p.); (3) LPS+fluoxetine (20 mg/kg, i.p.); (4) LPS+*F. aucheri* extract (100 mg/kg, i.p.); and (5) LPS+*F. aucheri* extract (200 mg/kg, i.p.). Lipopolysaccharide was administered to induce depressive-like behaviors, and after 24 hours, behavioral assessments were conducted using the forced swim test (FST), tail suspension test (TST), and open field test (OFT). Subsequently, immunohistochemical analysis was performed to assess the expression of toll-like receptor 4 (TLR4) and NF-κB in the brain tissue.

**Results:**

The FST and TST results revealed that treatment with *F. aucheri* extract significantly reduced immobility time compared to the LPS group, particularly at the 200 mg/kg dose, which showed superior efficacy even compared to fluoxetine. The OFT confirmed that the observed behavioral changes were not due to alterations in locomotor activity. Immunohistochemical analysis revealed that LPS significantly increased the expression of TLR4 and NF-κB in the brain. Notably, treatment with *F. aucheri* (200 mg/kg) significantly attenuated the expression of both biomarkers compared to the LPS group (#P < 0.05 and ##P < 0.01, respectively).

**Conclusions:**

The findings suggest that *F. aucheri* exhibits antidepressant-like effects in an LPS-induced model of depression, potentially mediated through modulation of neuroinflammatory pathways involving TLR4 and NF-κB. Given its promising preclinical efficacy and mechanistic relevance, *F. aucheri* could be considered an appropriate candidate for future clinical investigations as an antidepressant agent.

## 1. Background

Major depressive disorder (MDD) is one of the most prevalent and disabling psychiatric conditions worldwide, and it is projected to become the leading cause of disease burden by 2030 (1, 2). The etiology of MDD is multifactorial, involving complex interactions among genetic, developmental, and environmental factors that contribute to its heterogeneity (2). Despite the increasing number of available antidepressants, many individuals do not receive adequate treatment, and about half experience relapse even after treatment (3).

One of the key biological mechanisms implicated in the pathogenesis of depression is neuroinflammation. Social stress and systemic diseases such as diabetes, cancer, and cardiovascular conditions can increase the risk of depression, and many patients with MDD exhibit elevated levels of inflammatory markers (4-7). Several studies have reported increased expression of pro-inflammatory cytokines, including interleukin (IL)-1β, IL-6, and tumor necrosis factor-alpha (TNF-α), as well as acute phase proteins, in both the peripheral blood and cerebrospinal fluid (CSF) of individuals with depression (8-10). Elevated levels of key cytokines such as IL-1β, IL-6, and TNF-α can induce depressive-like behaviors by disrupting monoaminergic neurotransmission, impairing neuroplasticity, activating microglia, and enhancing oxidative and nitrosative stress, ultimately contributing to mood dysregulation and behavioral deficits (5, 11, 12). The toll-like receptor 4 (TLR4) signaling pathway, activated in response to bacterial endotoxins such as lipopolysaccharide (LPS), is a central mediator of immune activation in the brain and is strongly implicated in the development of depressive-like behaviors (13).

Lipopolysaccharide-induced depression in animal models mimics many of the inflammatory and behavioral aspects of MDD. Activation of TLR4 by LPS stimulates microglial cells to adopt a pro-inflammatory M1 phenotype, characterized by morphological changes and increased secretion of cytokines, nitric oxide (NO), reactive oxygen species (ROS), and other neurotoxic mediators (14). These changes contribute to impaired neuroplasticity, synaptic dysfunction, and reduced levels of brain-derived neurotrophic factor (BDNF), all of which are associated with depressive symptoms (15-17).

A pivotal intracellular signaling pathway that mediates inflammation and oxidative stress in this model is the TLR4/NF-κB cascade (18). Supporting the broader immunological relevance of this pathway, clinical evidence shows that bacterial lysates such as OM85-BV can significantly reduce systemic inflammatory markers (CRP, TNF-α, IL-6) and improve immune function in children with Streptococcus pneumoniae pulmonary infections through suppression of TLR-mediated NF-κB activation, highlighting the fundamental role of this pathway in human inflammatory diseases (19). Activation of nuclear factor kappa-light-chain-enhancer of activated B cells (NF-κB) leads to the transcription of numerous inflammatory genes and enzymes such as inducible nitric oxide synthase (iNOS), exacerbating neuroinflammation and oxidative damage (20). In addition to neuropsychiatric conditions, suppression of the TLR4/NF-κB pathway has also been shown to markedly attenuate inflammatory responses in non-neural disease models; for example, human umbilical cord mesenchymal stem cell–derived exosomes significantly reduced TLR4 and NF-κB expression as well as downstream cytokines (TNF-α, IL-6) in a rat model of acetic acid–induced proctitis, highlighting the broad relevance of this pathway in inflammation modulation (21). These opposing pathways represent a critical balance between oxidative damage and neuroprotection in depression.

Given the limitations and side effects associated with current antidepressant therapies, there is growing interest in plant-based treatments with antioxidant and anti-inflammatory properties (22, 23). *Ferula aucheri* (commonly known as Bilhar Kohi), a member of the Apiaceae family, is an endemic medicinal plant in Iran that is traditionally used to treat respiratory, gastrointestinal, and cardiovascular ailments (24). Phytochemical analyses have demonstrated that *F. aucheri* is rich in flavonoids, phenolic compounds, and terpenoids, which exhibit notable antioxidant and anti-inflammatory properties (25). Previous studies suggest that this plant may have hepatoprotective, hypolipidemic, and spasmolytic effects (26-28).

## 2. Objectives

Considering the similarities between *F. aucheri* and other medicinal plants that modulate inflammatory pathways — particularly through the inhibition of TLR4/NF-κB signaling — it may represent a novel candidate for treating inflammatory depression. *Ferula aucheri*, an endemic plant of Iran, contains abundant flavonoids, phenolic compounds, and terpenoids with potent antioxidant and anti-inflammatory activities (24-28). Although direct evidence of *F. aucheri* acting on TLR4 is limited, recent findings have demonstrated its ability to reduce systemic inflammatory mediators such as TNF-α and IL-6 while increasing anti-inflammatory cytokines like IL-10 (29), suggesting a possible influence on TLR4-associated pathways. In related studies, plant-derived polyphenols and terpenoids have been shown to suppress TLR4-mediated neuroinflammation and inhibit NF-κB activation, resulting in neuroprotective and antidepressant-like effects in LPS- or stress-induced models (30). Therefore, the present study aimed to evaluate the antidepressant-like effects of the hydroalcoholic extract of *F. aucheri* in a mouse model of LPS-induced depression and to investigate its possible mechanisms through modulation of TLR4 and NF-κB signaling pathways.

## 3. Methods

### 3.1. Preparation of Hydroalcoholic Extract of Ferula aucheri

In April 2023, we obtained specimens of *F. aucheri* with specimen code AR337E in the herbarium of the Biosciences Department of Shahid Beheshti University (Tehran) from Kermanshah Province, Iran. The plant material was air-dried at room temperature (25°C) in the absence of direct sunlight to preserve its phytochemical constituents. After drying, 1000 g of the plant material was ground into fine powder, and 300 g was extracted with a hydroalcoholic solvent mixture consisting of 800 mL of 97% ethanol and 400 mL of distilled water. The mixture was stirred continuously on a magnetic stirrer at room temperature for 48 hours. The extract was then filtered, and the solvent was removed under reduced pressure to obtain the total hydroalcoholic extract. After solvent removal, the dried extract was obtained with an approximate yield of 14% relative to the initial 300 g of plant powder.

### 3.2. Animals

Thirty male Swiss albino mice (8 weeks old, weighing 24 ± 3 g) were obtained from Elm Bavarian Aftab Company (Kermanshah, Iran). The animals were housed under standard laboratory conditions: temperature of 22 ± 2°C, 50 - 60% relative humidity, and a 12-hour light/dark cycle. Mice had free access to standard chow and purified water throughout the experiment. All experimental procedures were approved by the Institutional Animal Ethics Committee of Kermanshah University of Medical Sciences and were conducted in accordance with national ethical guidelines (Ethical no. IR.KUMS.REC.1400.567).

Thirty mice were randomized into five experimental groups (n = 6 per group) with an allocation ratio of 1:1:1:1:1 (control, LPS, LPS+fluoxetine (FLX), LPS+extract 100 mg/kg, LPS+extract 200 mg/kg). Randomization was performed using simple random number generation. Treatments were administered intraperitoneally (i.p.) to evaluate the potential antidepressant effects of *F. aucheri* extract at two different doses (100 and 200 mg/kg) (31) in comparison with fluoxetine (20 mg/kg) (30 min before LPS injection (1 mg/kg)). Lipopolysaccharide (LPS; *Escherichia coli* O111:B4; Sigma-Aldrich, USA) was freshly dissolved in sterile normal saline to prepare a 1 mg/mL stock solution on the day of administration. The solution was gently vortexed until fully dissolved and kept on ice, protected from light. The injection volume for each mouse was calculated based on body weight to ensure a final dose of 1 mg/kg administered intraperitoneally. Behavioral assessments were performed 24 hours after LPS administration (32). All animals were euthanized in accordance with the AVMA Guidelines for the Euthanasia of Animals (2020) using CO₂ inhalation, followed by cervical dislocation to ensure death, fully complying with institutional and national ethical standards (Table 1). 

**Table 1.** Description of Experimental Groups and Administered Doses in the Lipopolysaccharide-Induced Depression Model

Groups	Treatment Description	Dose (i.p.)
**Control**	Normal saline	1 mL/kg
**LPS**	LPS to induce depressive-like behavior	1 mg/kg
**LPS+FLX**	LPS followed by fluoxetine (standard antidepressant)	LPS: 1 mg/kg+fluoxetine: 20 mg/kg
**LPS+Extract 100 mg/kg**	LPS followed by Ferula aucheri extract (100 mg/kg)	LPS: 1 mg/kg+extract: 100 mg/kg
**LPS+Extract 200 mg/kg**	LPS followed by Ferula aucheri extract (200 mg/kg)	LPS: 1 mg/kg+extract: 200 mg/kg

Abbreviations: LPS, lipopolysaccharide; FLX, fluoxetine.

### 3.3. Behavioral Assessments

#### 3.3.1. Forced Swim Test

Each mouse was placed individually in a transparent glass cylinder (25 cm height, 10 cm diameter) filled with water (25 ± 1°C) to a depth of 15 cm. The total immobility time was recorded over four 5-minute test sessions, each separated by a 90-second rest period. Immobility was defined as the absence of active escape-directed behaviors, indicating depressive-like behavior (33).

#### 3.3.2. Tail Suspension Test

Mice were suspended by the tail using adhesive tape placed approximately 1 cm from the tip, attached to a horizontal bar 50 cm above the bench. The total immobility time was recorded during four 5-minute intervals, with 90-second rest periods between each session. Immobility was interpreted as a behavioral correlate of despair (34).

#### 3.3.3. Open Field Test

Each mouse was placed in the center of a circular open-field arena (50 cm × 50 cm) and its activity was monitored for 5 minutes. Parameters measured included the number of movements and the time spent in the central zone. The open field test (OFT) was used to confirm that changes in immobility were not due to altered locomotor activity (35).

### 3.4. Immunohistochemistry for NF-κB and Toll-Like Receptor 4 Expression

Following behavioral testing, mice were anesthetized with a combination of ketamine and xylazine and euthanized. Brains were rapidly removed, washed with cold saline, and fixed in formalin. Tissue sections (5 µm) were prepared and subjected to immunohistochemical staining using primary antibodies specific for NF-κB and TLR4: Anti-NF-κB p65 antibody (ab16502); anti-TLR4 (sc-293072). Secondary antibodies were applied according to the manufacturer’s instructions, and immunoreactivity was visualized under a light microscope (Fluorescent and light microscope (Germany-AXIOM)). The optical density of positively stained regions was quantified using ImageJ software (36).

### 3.5. Statistical Analysis

All data were analyzed using GraphPad Prism version 9.0 (GraphPad Software, San Diego, CA, USA). Before analysis, data were examined for outliers using the ROUT test (Q = 1%) and were tested for normality using the Shapiro-Wilk test and for homogeneity of variances using Levene’s test. Behavioral outcomes [forced swim test (FST), tail suspension test (TST), and OFT parameters] and immunohistochemistry optical density values were normally distributed and were therefore expressed as mean ± standard error of the mean (SEM). Statistical differences among groups were evaluated using one-way analysis of variance (one-way ANOVA) followed by Tukey’s multiple comparison post hoc test to determine pairwise differences between treatment groups. For all analyses, a P-value < 0.05 was considered statistically significant. In all graphical representations, significance levels were indicated as * P < 0.05, ** P < 0.01, and *** P < 0.001.

## 4. Results

### 4.1. Effects of Different Doses of Ferula aucheri Extract in Lipopolysaccharide-Induced Depression Model in the Forced Swim Test

**Figure 1.**
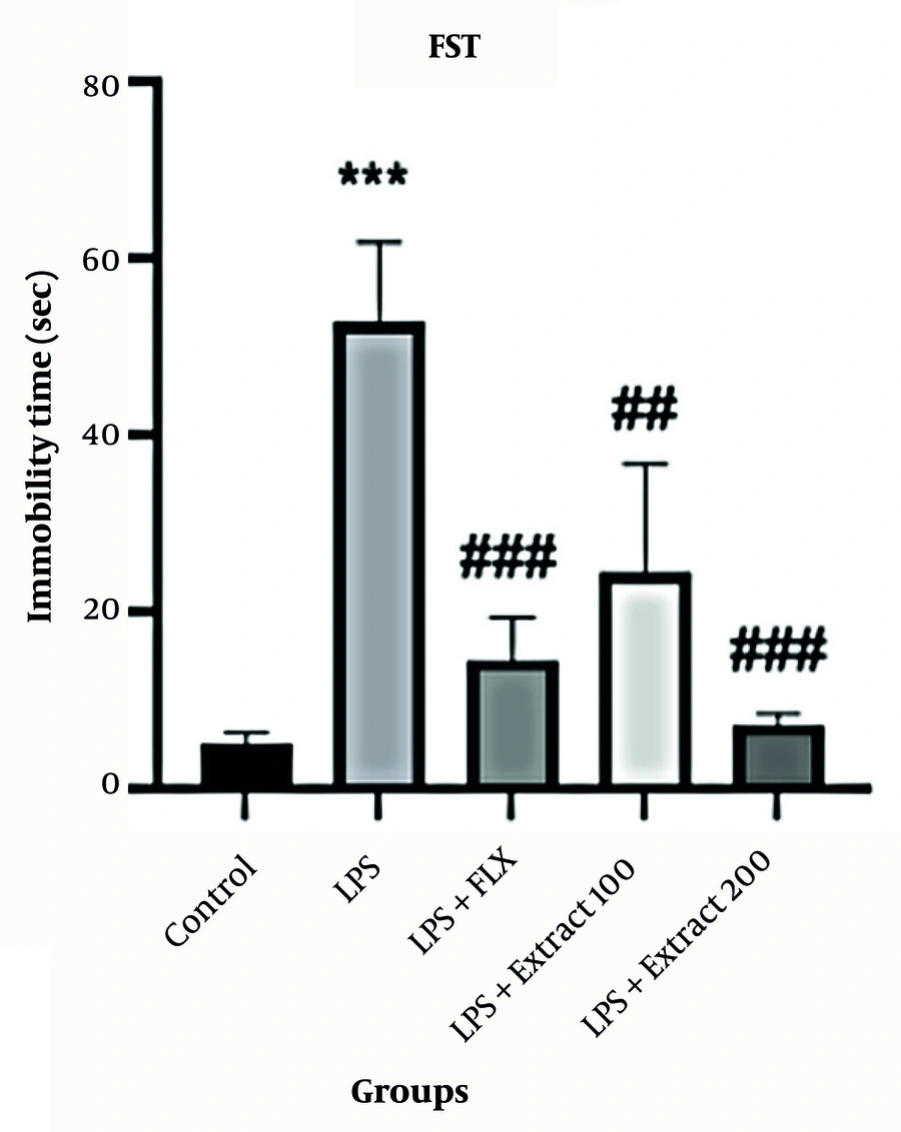
Effect of different doses of *Ferula aucheri* extract on immobility time in lipopolysaccharide (LPS)-induced depression model in the forced swim test (FST); this figure illustrates the effects of different doses of *F. aucheri* hydroalcoholic extract (100 and 200 mg/kg) and fluoxetine (20 mg/kg) on immobility time in mice subjected to LPS-induced depressive-like behavior. Lipopolysaccharide administration markedly increased immobility time compared with the control group (*** P < 0.001). Treatment with fluoxetine significantly reduced immobility time (### P < 0.001 vs. LPS). Both doses of the extract significantly decreased immobility time relative to the LPS group, with the 200 mg/kg dose showing the most pronounced effect (### P < 0.001 vs. LPS). Data are presented as mean ± SEM.

The immobility time in the FST is shown in Figure 1. Lipopolysaccharide administration significantly increased immobility time compared to the control group (*** P < 0.001; 55 ± 8 s vs. 5 ± 2 s). Treatment with the standard antidepressant fluoxetine (20 mg/kg) significantly reduced immobility time compared to the LPS group (### P < 0.001; 17 ± 4 s). Moreover, the hydroalcoholic extract of *F. aucheri* at doses of 100 and 200 mg/kg significantly reduced the immobility time in comparison to the LPS group (## P < 0.01, ### P < 0.001; 25 ± 10 sec and 10 ± 3 s, respectively). Notably, the 200 mg/kg dose demonstrated the most pronounced effect, showing a greater reduction in immobility time than both the LPS and fluoxetine groups (### P < 0.001).

### 4.2. Effects of Different Doses of Ferula aucheri Extract in Lipopolysaccharide-Induced Depression Model in the Tail Suspension Test

**Figure 2.**
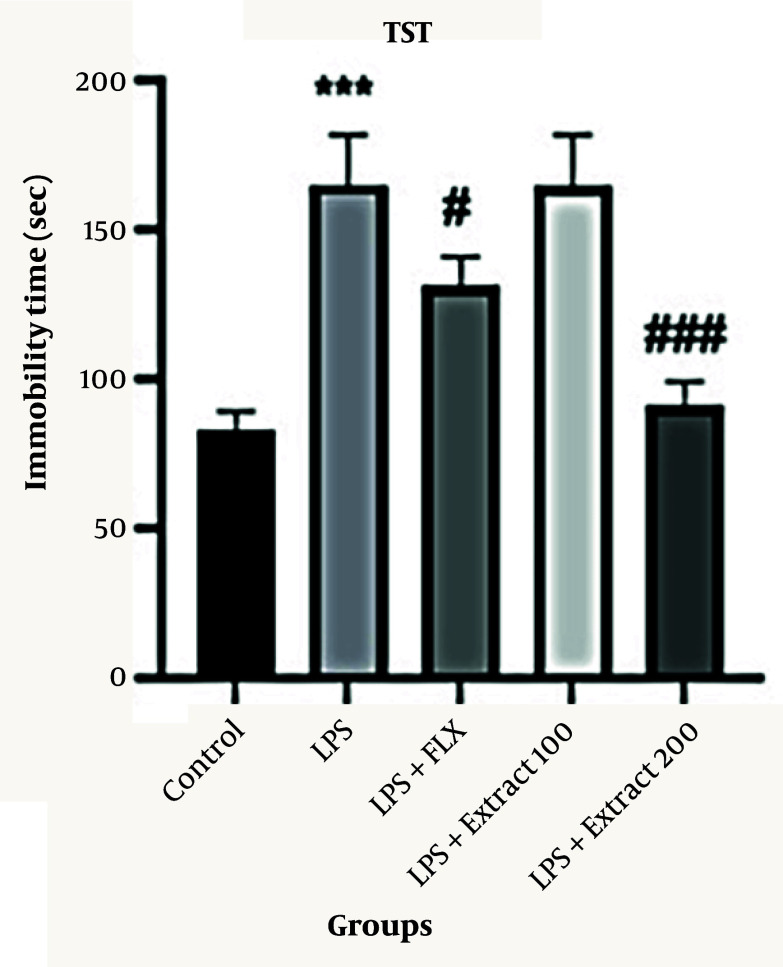
Effect of different doses of *Ferula aucheri* extract on immobility time in lipopolysaccharide (LPS)-induced depression model in the tail suspension test (TST); this figure shows the impact of *F. aucheri* hydroalcoholic extract (100 and 200 mg/kg) and fluoxetine (20 mg/kg) on immobility time in the LPS-induced depression model using the TST. Lipopolysaccharide administration markedly increased immobility time compared with the control group (*** P < 0.001). Fluoxetine significantly reduced immobility duration compared to the LPS group (# P < 0.05). The 200 mg/kg dose of the extract produced a robust antidepressant-like effect, significantly decreasing immobility time relative to LPS (### P < 0.001), while the 100 mg/kg dose showed a moderate but nonsignificant reduction. Data are presented as mean ± SEM.

As shown in Figure 2, mice in the LPS group exhibited a significantly increased immobility time in the TST compared to the control group (*** P < 0.001; 165 ± 12 s vs. 80 ± 10 s). Treatment with fluoxetine significantly reduced immobility time compared to the LPS group (# P < 0.05; 135 ± 10 s), although this reduction did not reach statistical significance when compared with the control group. Administration of *F. aucheri* extract at 200 mg/kg significantly decreased immobility time compared to the LPS group (### P < 0.001; 95 ± 10 s). In contrast, the 100 mg/kg dose did not produce a significant reduction relative to either the LPS group or the control group, with immobility time remaining elevated (P > 0.05, 160 ± 15 s).

### 4.3. Effects of Ferula aucheri Extract on Locomotor Activity in the Open Field Test

**Figure 3.**
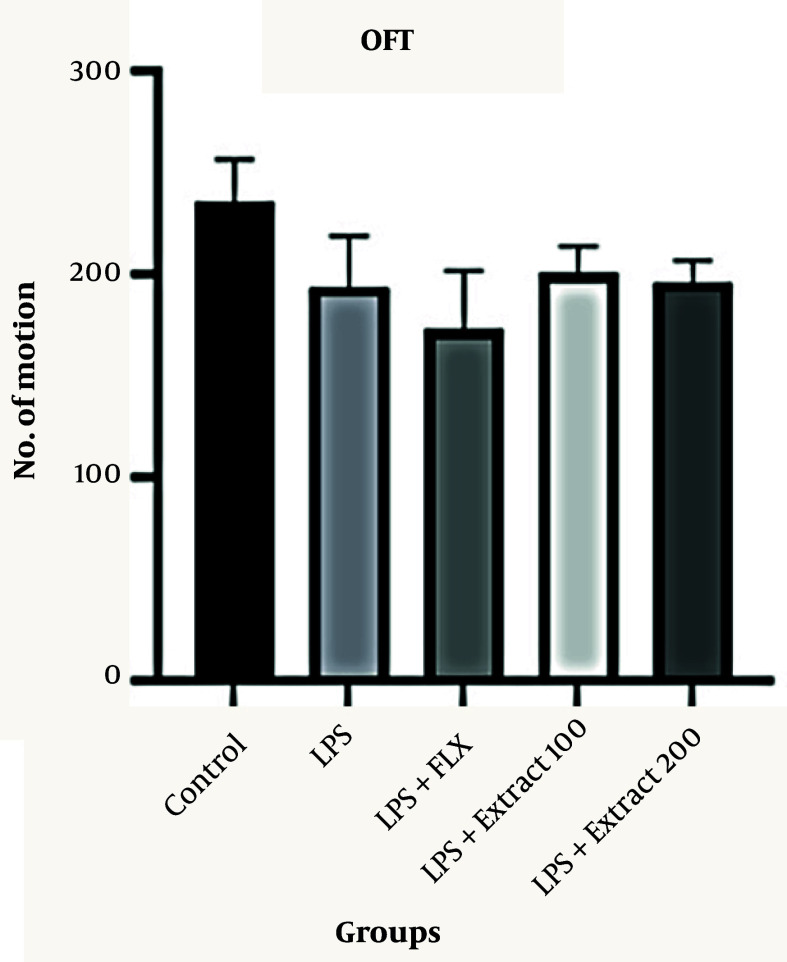
Evaluation of locomotor activity using the open field test (OFT); this figure illustrates the locomotor activity of mice in the OFT following treatment with *Ferula aucheri* extract (100 and 200 mg/kg), fluoxetine (20 mg/kg), or lipopolysaccharide (LPS) alone. The total number of movements did not differ significantly among the groups (P > 0.05), indicating that neither LPS nor the treatments affected baseline locomotor behavior. These findings confirm that changes observed in the forced swim test (FST) and tail suspension test (TST) were not influenced by alterations in motor activity. Data are presented as mean ± SEM.

To rule out locomotor dysfunction as a confounding factor in behavioral tests, the OFT was performed. The results revealed no significant differences in locomotor activity among the groups (P > 0.05), with locomotor counts recorded as follows: Control: 240 ± 15, LPS: 195 ± 15, LPS+FLX: 180 ± 20, extract 100 mg/kg: 200 ± 18, and extract 200 mg/kg: 195 ± 15 (Figure 3). These findings confirm that the observed changes in FST and TST are not due to altered motor activity but rather reflect mood-related behavioral changes.

### 4.4. Immunohistochemical Findings

#### 4.4.1. Toll-Like Receptor 4 Expression

**Figure 4.**
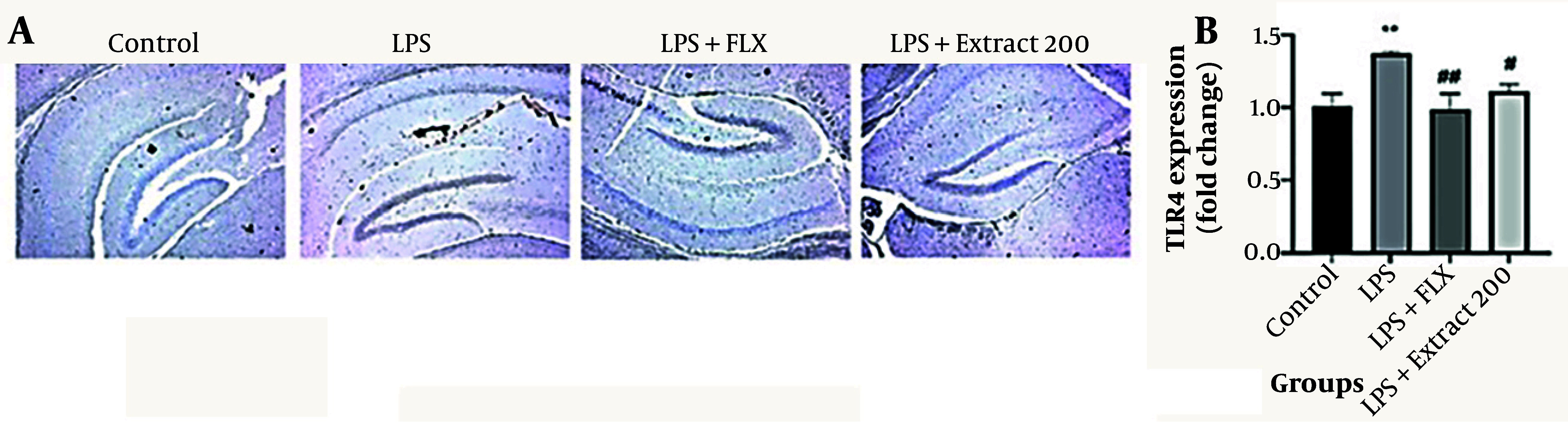
Effects of *Ferula aucheri* extract on toll-like receptor 4 (TLR4) expression in lipopolysaccharide (LPS)-induced neuroinflammation; this figure presents the immunohistochemical evaluation of TLR4 expression in the hippocampal region of mice across experimental groups. A, representative coronal brain sections showing TLR4 immunostaining in the control, LPS, LPS+fluoxetine, and LPS+*F. aucheri* extract (200 mg/kg) groups. Lipopolysaccharide administration markedly increased TLR4-positive staining compared with the control group. Both fluoxetine and the plant extract reduced TLR4 immunoreactivity, with the extract demonstrating a noticeable attenuation of TLR4 expression; B, quantitative analysis of TLR4 expression levels (fold change). Lipopolysaccharide significantly upregulated TLR4 expression (** P < 0.01 vs. control). Fluoxetine administration resulted in a significant reduction (## P < 0.01 vs. LPS), and treatment with *F. aucheri* extract at 200 mg/kg also decreased TLR4 levels (# P < 0.05 vs. LPS). Data are expressed as mean ± SEM.

Immunohistochemical analysis of brain sections (Figure 4) showed a significant upregulation of TLR4 expression in the LPS-treated group compared to controls (** P < 0.01; 1.35 ± 0.07 vs. 1.00 ± 0.10). Fluoxetine administration significantly downregulated TLR4 levels compared to the LPS group (## P < 0.01; 1.05 ± 0.05). Treatment with *F. aucheri* extract at 200 mg/kg also reduced TLR4 expression compared to the LPS group (# P < 0.05; 1.15 ± 0.05), although the magnitude of reduction was less pronounced than that observed with fluoxetine.

#### 4.4.2. NF-κB Expression

**Figure 5.**
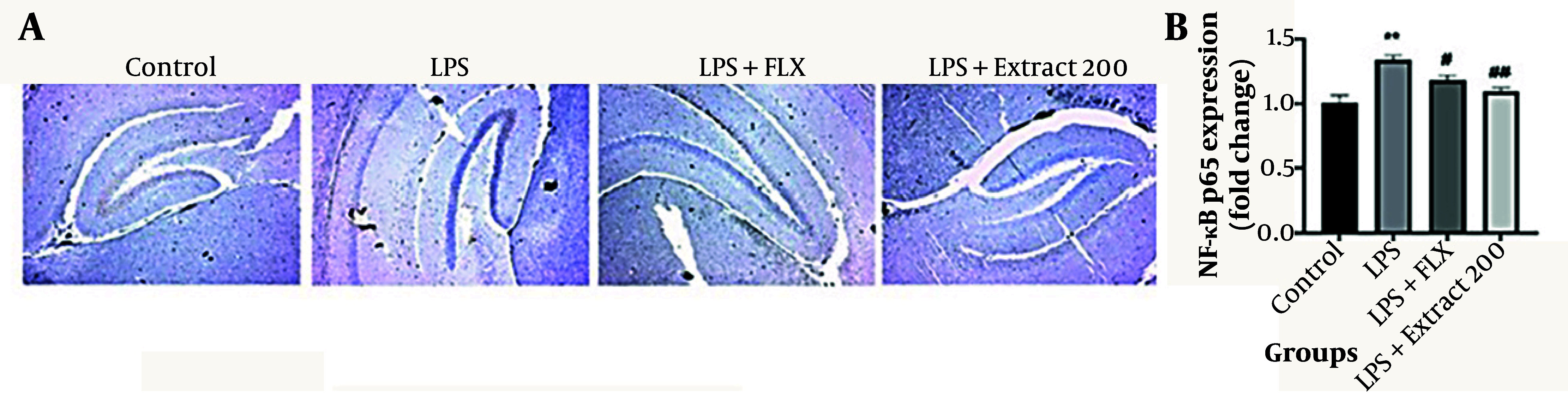
Effects of *Ferula aucheri* extract on NF-κB p65 expression in lipopolysaccharide (LPS)-induced neuroinflammation; this figure illustrates the immunohistochemical evaluation of NF-κB p65 expression in the hippocampal region across experimental groups. A, representative brain sections showing NF-κB immunostaining in the control, LPS, LPS+fluoxetine, and LPS+*F. aucheri* extract (200 mg/kg) groups. Lipopolysaccharide markedly increased NF-κB-positive staining compared to the control group, indicating activation of pro-inflammatory signaling. Both fluoxetine and the plant extract visibly reduced NF-κB immunoreactivity, with the extract demonstrating stronger attenuation; B, quantitative analysis of NF-κB p65 expression (fold change); LPS treatment significantly upregulated NF-κB levels (** P < 0.01 vs. control). Administration of fluoxetine reduced NF-κB expression (# P < 0.05 vs. LPS), and *F. aucheri* extract (200 mg/kg) produced an even greater reduction (## P < 0.01 vs. LPS). Data are presented as mean ± SEM.

As illustrated in Figure 5, LPS treatment significantly increased NF-κB expression in brain tissues compared to the control group (** P < 0.01; 1.30 ± 0.05 vs. 1.00 ± 0.10). Administration of *F. aucheri* extract at 200 mg/kg significantly reduced NF-κB expression compared to the LPS group (## P < 0.01; 1.05 ± 0.05), indicating its potential anti-inflammatory effects at the molecular level. Fluoxetine also attenuated NF-κB expression, though to a lesser extent than the extract (# P < 0.05, 1.10 ± 0.05).

## 5. Discussion

Major depression (MD) is a prevalent and disabling psychiatric disorder and is projected to become the leading cause of global disease burden by 2030 (37). Despite the widespread use of antidepressants, their efficacy remains suboptimal, with a high rate of relapse and treatment resistance in many patients (23, 38). Comorbid conditions such as diabetes, cancer, and cardiovascular disease significantly increase depression risk, underscoring the role of systemic inflammation in its pathogenesis (39-41).

Substantial evidence indicates that neuroinflammation plays a pivotal role in depression, with increased levels of pro-inflammatory cytokines (IL-1β, IL-6, TNF-α), acute-phase reactants, and Toll-like receptors such as TLR4 observed in both clinical and postmortem studies of depressed individuals (11, 42, 43). Experimental models using LPS, a potent TLR4 agonist, consistently replicate depressive-like behaviors and are widely employed in preclinical antidepressant screening (44-46). LPS activates microglia, promoting the expression of inflammatory transcription factors, such as NF-κB, which further amplifies immune responses and induces depressive phenotypes (47-50).

In the present study, administration of *F. aucheri* extract significantly alleviated LPS-induced depressive-like behavior in both the FST and TST. Among the tested doses, only the 200 mg/kg extract produced a robust and statistically significant antidepressant-like effect, while fluoxetine and the 100 mg/kg extract showed partial but non-significant reductions in immobility compared to the control group. These findings are consistent with prior studies reporting antidepressant-like effects of herbal agents, such as Carthamus tinctorius (51), Acorus calamus (52), and Citrus maxima (53), which are often attributed to monoamine oxidase inhibition or modulation of neurotransmission.

Notably, the OFT results confirmed that reductions in immobility were not due to impaired locomotor activity, supporting the specificity of the antidepressant-like effects. This aligns with other herbal-based antidepressant models, including those involving *Onosma bracteatum* (51) and *Verbena officinalis* (52), which similarly showed improvements in exploratory behavior without affecting basal activity.

At the molecular level, immunohistochemistry revealed that *F. aucheri* extract (200 mg/kg) significantly downregulated TLR4 and NF-κB expression in the brain compared to mice treated with LPS. Although fluoxetine also reduced the expression of these inflammatory markers, its behavioral effects in this study were less pronounced compared to the 200 mg/kg extract. Supporting the relevance of NF-κB inhibition in inflammatory disorders, ketotifen has also been shown to markedly reduce NF-κB expression, alongside pro-inflammatory cytokines (TNF-α, IL-6, IL-1β) and oxidative stress markers, in a gentamicin-induced hepatotoxicity model. This demonstrates that suppression of this pathway confers broad anti-inflammatory and tissue-protective effects (53). This pattern is consistent with phytocompounds such as sinomenine (48), icariin (49), and arctigenin (50), which exert antidepressant effects by inhibiting the TLR4/NF-κB pathway

Phytochemical studies indicate that *F. aucheri* contains several bioactive constituents — including flavonoids, phenolic acids, coumarins, sesquiterpene lactones, and terpenoids — that exhibit potent antioxidant and anti-inflammatory properties (24, 26, 28). Many of these metabolites, particularly flavonoids and polyphenols, have been shown to modulate key neurobiological mechanisms implicated in depression. Flavonoid- and polyphenol-rich preparations can suppress pro-inflammatory cytokines, inhibit microglial activation, attenuate oxidative stress, and modulate monoaminergic neurotransmission and HPA axis activity (23, 54). Moreover, several phytochemicals structurally related to those reported in *Ferula* species — such as icariin, arctigenin, and gypenosides — exert antidepressant-like effects by inhibiting the TLR4/MyD88/NF-κB signaling cascade (30, 49, 50), which is consistent with the pathway targeted in the present LPS-induced depression model. In addition, recent pharmacological data on *F. aucheri* have demonstrated systemic anti-inflammatory activity, including reductions in TNF-α and IL-6 and increases in IL-10 and HSP-70 levels (29), further supporting the notion that the observed behavioral effects are mediated, at least in part, through the combined anti-inflammatory and antioxidant actions of its constituent compounds.

Importantly, recent studies further reinforce these mechanisms. A survey by Noori et al. demonstrated that Citrus medica extract, like *F. aucheri*, reduced depressive behavior by suppressing iNOS and pro-inflammatory cytokines in an LPS-induced mouse model (32). Similarly, Mohamed et al. reported that trimetazidine, an anti-ischemic agent, ameliorated LPS-induced depression by inhibiting TLR4/NF-κB and activating the Nrf2/HO-1 antioxidant pathway (20). These findings support the hypothesis that dual anti-inflammatory and antioxidant modulation may underlie the observed effects of *F. aucheri*.

Additional evidence from a study on *F. aucheri* in wound healing revealed reductions in TNF‑α and IL‑6 and increases in IL‑10 and HSP-70, confirming the plant’s potent systemic anti-inflammatory activity (29). Furthermore, a study on gypenosides demonstrated that shifting microglial polarization from M1 to M2 by inhibiting the TLR4/MyD88/NF-κB axis reduced IL-1β and IL-6 levels and improved behavioral outcomes in depression models (30). In addition, asperosaponin VI has been shown to suppress the TLR4/NF-κB/IDO axis and modulate glutamatergic signaling in depression (28), supporting the possibility of a similar mechanism in *F. aucheri*. Flavonoids, abundant in *F. aucheri*, are also known to regulate neurotransmitters and the HPA axis beyond their anti-inflammatory effects (54).

In addition to its anti-inflammatory actions, several complementary pharmacological mechanisms may further explain the antidepressant-like effects observed in this study. Chronic inflammation and LPS exposure are known to impair neuroplasticity and reduce brain-derived neurotrophic factor (BDNF), whereas flavonoids and terpenoids present in *Ferula* species have been associated with enhanced BDNF signaling and improved synaptic resilience (15, 37). Moreover, oxidative stress is a key mediator of microglial overactivation in depression, and the strong antioxidant properties of polyphenolic constituents can attenuate ROS accumulation and restore redox homeostasis (54). The extract may also indirectly modulate dysregulation of the hypothalamic-pituitary-adrenal (HPA) axis, a central pathway in stress-related depressive disorders (37). These convergent mechanisms provide additional biological plausibility for the robust behavioral efficacy observed at the 200 mg/kg dose.

The central role of the TLR4/NF-κB signaling axis in neuroimmune regulation and the pathophysiology of depression has been demonstrated across various models. Interventions such as Xiao-Chai-Hu-Tang (XCHT) (55), oregano essential oil (OEO) (56), and Kai-Xin-San (KXS) (57) have also shown efficacy in targeting this pathway, supporting the therapeutic potential of anti-inflammatory botanicals.

While the findings provide compelling evidence for the antidepressant-like effects of *F. aucheri*, several limitations must be acknowledged. One methodological limitation relates to the use of whole-brain coronal sections rather than region-specific analyses. Whole-brain immunohistochemistry was employed because the tissue processing workflow was originally designed to evaluate global neuroinflammatory activation, and region-specific microdissection (such as hippocampus or frontal cortex isolation) was not technically feasible at the time of sampling. Although whole-brain immunohistochemistry is an accepted initial screening method in LPS-induced neuroinflammation models (14, 15), future studies should incorporate region-specific assessments to provide more detailed insights into the neural circuits involved. Furthermore, this study did not explore the role of antioxidant pathways such as Nrf2/HO-1, which may also contribute to the extract’s effects. Moreover, the present study utilized the total hydroalcoholic extract rather than fractionated extracts because this work served as an initial pharmacological screening. Whole-extract preparations preserve the natural synergy among flavonoids, terpenoids, coumarins, and phenolic compounds, which may collectively contribute to the antidepressant effect. Future investigations should include bioactivity-guided fractionation to identify the most active constituents and compare the antidepressant efficacy of individual fractions. No toxicity or safety profile was assessed for the extract, although no overt adverse effects were observed at the tested doses. Third, only male mice were used, and sex-based differences in inflammatory response and depression have been reported. Finally, protein-level validation via Western blot or ELISA could strengthen the mechanistic conclusions drawn from immunohistochemistry alone.

Future studies should assess the safety profile and pharmacokinetics of *F. aucheri* extract. Investigating its interaction with conventional antidepressants may also clarify its potential as an adjunct therapy. Additionally, mechanistic exploration of Nrf2/HO-1, MAO activity, and neurotransmitter modulation will provide a more comprehensive understanding of its antidepressant mechanisms. Ultimately, clinical trials will be needed to validate its therapeutic efficacy in humans.

Collectively, these findings support the traditional use of *F. aucheri* and demonstrate its dual behavioral and anti-inflammatory effects in a validated model of inflammatory depression. This contributes to the growing body of evidence supporting the development of phytotherapeutics that target neuroimmune signaling.

### 5.1. Conclusions

The present study provides strong evidence that *F. aucheri* hydroalcoholic extract possesses notable antidepressant-like effects in an inflammation-induced murine model of depression, primarily through suppression of the TLR4/NF-κB signaling pathway. Its efficacy at 200 mg/kg, without impairing locomotion, highlights its targeted behavioral benefit. Compared to fluoxetine, *F. aucheri* exhibited superior inhibition of NF-κB, suggesting therapeutic promise as either an alternative or adjunct treatment. Supported by recent findings on similar natural compounds and their documented anti-inflammatory, antioxidant, and phenolic properties, *F. aucheri* emerges as a credible candidate for phytotherapy in depression. Further research is warranted to explore its broader molecular effects, validate protein-level changes, and establish long-term safety across sexes.

## Data Availability

The dataset presented in the study is available on request from the corresponding author during submission or after publication.
